# A Mendelian Randomization Study of Plasma Homocysteine Levels and Cerebrovascular and Neurodegenerative Diseases

**DOI:** 10.3389/fgene.2021.653032

**Published:** 2021-04-01

**Authors:** Weishi Liu, Luyang Zhang, Shen Li, Chen Liu, Ying Tong, Hui Fang, Rui Zhang, Bo Song, Zongping Xia, Yuming Xu

**Affiliations:** ^1^Department of Neurology, The First Affiliated Hospital of Zhengzhou University, Zhengzhou, China; ^2^Henan Key Laboratory of Cerebrovascular Diseases, The First Affiliated Hospital of Zhengzhou University, Zhengzhou, China; ^3^Clinical Systems Biology Laboratories, The First Affiliated Hospital of Zhengzhou University, Zhengzhou, China

**Keywords:** Mendelian randomization, homocysteine, cerebrovascular disease, neurodegenerative disease, small artery occlusion, blood pressure, diabetes mellitus

## Abstract

**Background**: Homocysteine (Hcy) is a toxic amino acid and hyperhomocysteinemia (HHcy) was reported to be associated with both cerebrovascular disease and neurodegenerative disease. Our aim was to assess the causal link between plasma Hcy level and cerebrovascular and neurodegenerative diseases through a Mendelian randomization (MR) study.

**Methods**: A two-sample MR study was performed to infer the causal link. We extracted the genetic variants (SNPs) associated with plasma Hcy level from a large genome-wide association study (GWAS) meta-analysis. The main MR analysis was performed using the inverse variance-weighted method. Additional analyses were further performed using MR-Egger intercept and Cochran’s Q statistic to detect the heterogeneity or pleiotropy of our findings.

**Results**: Thirteen Hcy-associated SNPs were selected as instrumental variables. The results showed evidence of a causal link between plasma Hcy level and ischemic stroke (IS) caused by small artery occlusion (SAS, OR = 1.329, 95% CI 1.047–1.612, *p* = 0.048). Meanwhile, there was no evidence of association between plasma Hcy level and other types of IS, transient ischemic attack (TIA), or neurodegenerative disease. The MR-Egger intercept test indicated no evidence of directional pleiotropy. Results of additional MR analysis indicated that blood pressure (BP) and type 2 diabetes mellitus (T2DM) serve as influencers in the association.

**Conclusion**: The MR study found a little causal link between plasma Hcy level and SAS. The link is likely to be influenced by other risk factors like BP and T2DM.

## Introduction

Homocysteine (Hcy) is a sulfur-containing toxic amino acid that is harmful to the body or cells (Eikelboom et al., 1999; Faraci and Lentz, 2004). Hcy has been shown to induce endothelial dysfunction by DNA damage, oxidative stress, and promotion of coagulation (Hankey and Eikelboom, 2001). In addition, hyperhomocysteinemia (HHcy) also adversely affects vascular smooth muscle cells, leading to their proliferation (Mujumdar et al., 2000). Therefore, the toxicity of Hcy is considered a cause of vascular alterations and atherosclerosis. Hcy has been regarded as a risk factor for cardiovascular disease since 1969 (McCully, 1969; Eikelboom et al., 1999). However, lowering Hcy medications, including folate and B vitamin supplementation, remains limited as most clinical trials have shown conflicting results (Huang et al., 2012). Although studies have reported that lowering Hcy may reduce the risk of stroke (Martí-Carvajal et al., 2017), the utilization of lowering Hcy medication is not well determined based on the subtypes of ischemic stroke (IS), as demonstrated by The Trial of Org 10,172 in Acute Stroke Treatment (TOAST), including large artery atherosclerosis (LAS), cardioembolism (CES), and small artery occlusion (SAS; Adams et al., 1993).

In addition to cerebrovascular disease, HHcy was reported to be associated with other neurological disorders, especially neurodegenerative disease (Mattson and Shea, 2003; Dubchenko et al., 2020). Neurodegeneration is characterized by neuronal degeneration and apoptosis. Previous studies have reported the association of HHcy and Alzheimer’s disease (AD), Parkinson’s disease (PD), and amyotrophic lateral sclerosis (ALS; Seshadri et al., 2002; Wang and Fan, 2012; Fan et al., 2020). In addition, vascular origin was mentioned as a potential mechanism of neurodegeneration and demyelination (Kalaria et al., 2012; Zivadinov et al., 2012; Yamazaki and Kanekiyo, 2017). It is still unclear whether HHcy is the cause or merely a phenomenon accompanying cardiovascular disease.

Because of sampling errors, causal links between plasma Hcy level and risk of cerebrovascular or neurodegenerative disease cannot be found by observational studies. Mendelian randomization (MR) is a powerful tool for analyzing the causality of exposure factors and certain disorder, which utilizes genetic variations (i.e., SNPs, single nucleotide polymorphisms) as instrumental variables (IVs; Lawlor et al., 2008; Sekula et al., 2016). Thus, an MR study can overcome the limitations of observational studies such as confounding and reverse causation (Latvala and Ollikainen, 2016). Previous studies have investigated the causal link between Hcy and cardiovascular disease or AD, but few focused on the subtypes of IS or other neurodegenerative disorders (Casas et al., 2005; Larsson et al., 2017; Miao et al., 2019).

The aim of our study was to evaluate the causal association between plasma Hcy level and cerebrovascular disease, including IS (LAS, CES, SAS, and nonsubtyped) and transient ischemic attack (TIA), or neurodegenerative disease, including MS, AD, PD, ALS, and frontotemporal dementia (FTD) through a two-sample MR study.

## Materials and Methods

### Data Sources

All the genetic variants associated with plasma Hcy level were acquired from a large genome-wide association study (GWAS) meta-analysis with 44,147 subjects of European ancestry (van Meurs et al., 2013). For the cerebrovascular disease dataset, we obtained the corresponding genetic variants from the MEGASTROKE consortium (Malik et al., 2018). Their dataset included 440,328 subjects and 34,217 cases, which could be further divided into LAA (*n* = 4,373), CE (*n* = 7,193), small artery occlusion (SAO; *n* = 5,386), and nonsubtyped cases (*n* = 17,265; Malik et al., 2018). We obtained corresponding genetic variants with TIA from the UK Biobank, including 1,364 cases with TIA and 461,646 controls (Sudlow et al., 2015). For datasets of neurodegenerative disease, we obtained the corresponding genetic variants from the International Multiple Sclerosis Genetics Consortium (IMSGC) including 14,498 cases with MS and 24,091 controls [International Multiple Sclerosis Genetics Consortium (IMSGC) et al., 2013], the International Genomics of Alzheimer’s Project including 21,982 cases with AD and 41,944 controls (Kunkle et al., 2019), the International Parkinson’s Disease Genomics Consortium including 33,674 cases with PD and 449,056 controls (Nalls et al., 2019), the International Amyotrophic Lateral Sclerosis Genomics Consortium including 20,806 cases with ALS and 59,804 controls (Nicolas et al., 2018), and the International Frontotemporal Lobar Degeneration Collaboration including 515 cases with FTD and 2,509 controls (Van Deerlin et al., 2010). The subjects from both exposure and outcome datasets included in our study were of European ancestry. Ethics approval was not required as it was a secondary analysis of previously published data.

### Study Design

**Figure 1:**
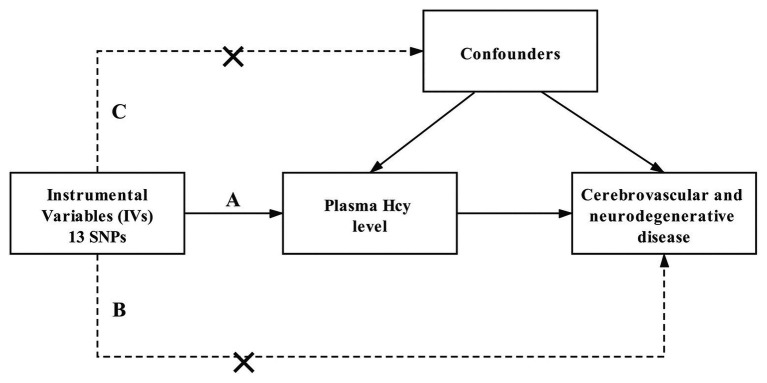
Design and main assumptions of our Mendelian randomization study. SNPs, single nucleotide polymorphisms; Hcy, homocysteine.

A two-sample MR study was performed to investigate the potential causal impact of plasma Hcy level on the risk of cerebrovascular and neurodegenerative diseases. The MR study is established by three major assumptions (Emdin et al., 2017; Figure 1). First, the IVs are directly associated with the exposure (plasma Hcy level) with genome-wide significance. Second, there is no link between the IVs and the confounding factors. Lastly, the IVs affect the outcome merely through exposure.

### SNP Selection and Validation

We have selected genome-wide significant (*p* < 5 × 10^−8^) genetic variants (SNPs) associated with plasma Hcy level from a large GWAS meta-analysis as IVs (van Meurs et al., 2013). There were 18 SNPs in total, after testing for linkage disequilibrium: 5 SNPs (rs7422339, rs12134663, rs957140, rs12921383, and rs2851391) with *r*^2^ greater than 0.01 in the European samples of 1,000 Genomes were excluded and finally 13 SNPs remained (Supplementary Table S1). The 13 unique SNPs explained 5.9% of the variation in plasma Hcy level (van Meurs et al., 2013). Then, we have assessed the *F*-statistic of the selected IVs that was approximately 212.85, indicating strong instruments for our following MR study (Burgess et al., 2011). If the specified SNP was not available in the outcome dataset, we used a proxy SNP in linkage disequilibrium (*r*^2^ > 0.9) with the specified SNP. For the outcome dataset of TIA, MS, and FTD, there were no corresponding SNPs from the dataset, and thus, we would select a proxy variant with the existence of high linkage disequilibrium for further MR analysis.

### Statistical Analysis

MR analysis was conducted in R (version 4.0.2) by the TwoSampleMR package (Hemani et al., 2018). The main analysis was performed by random-effects inverse variance-weighted (IVW) analysis (Hemani et al., 2018). We also performed fixed-effects IVW analysis, maximum likelihood analysis, simple median analysis, MR-Egger analysis, weighted median analysis, simple mode analysis, and weighted mode analysis as additional analyses (Bowden et al., 2015, 2016; Hartwig et al., 2017). Then, sensitivity tests were conducted using the heterogeneity test, pleiotropy test, and leave-one-out sensitivity test. Cochran’s Q test was calculated to assess the degree of heterogeneity across the individual effect estimates derived from every genetic variant. The MR-Egger intercept test was conducted to assess the horizontal pleiotropy and a funnel plot was plotted to provide a visual inspection (Bowden et al., 2015). Leave-one-out sensitivity analysis was performed to measure if the pooled estimate is being disproportionately influenced by each genetic variant. We used a Bonferroni correction [corrected *p* = 0.05/1 (traits considered)/10 (exposures) = 0.005] to account for multiple comparisons.

In the leave-one-out analysis of SAS (Supplementary Figure S1), we found six IVs (rs154657, rs7130284, rs234709, rs42648, rs1801222, and rs2275565) which had a greater impact on the result. In addition, by the forest plot (Supplementary Figure S2), we found three potential risk SNPs (rs9369898, rs548987, and rs1801133) for SAS through the impact on plasma Hcy level. Therefore, we performed an additional MR analysis for the three and seven SNPs (in addition to the six SNPs mentioned above) and SAS.

The other additional MR analysis was first performed to find the 13 SNPs which showed significant differences for the known risk factors of IS (here considered as potential pleiotropic SNPs), including body mass index (BMI; Hoffmann et al., 2018), years of schooling (Lee et al., 2018), numbers of moderate physical activity, alcohol consumption (Clarke et al., 2017), current smoking, high-density lipoprotein cholesterol (HDL; Willer et al., 2013), low-density lipoprotein cholesterol (LDL; Willer et al., 2013), total cholesterol (TC; Kettunen et al., 2016), triglycerides (TG; Willer et al., 2013), pulse rate (PR), systolic blood pressure (SBP; Evangelou et al., 2018), diastolic blood pressure (DBP; Evangelou et al., 2018), atrial fibrillation (AF; Nielsen et al., 2018), coronary heart disease (CHD; Nikpay et al., 2015), fasting blood glucose (FBG; Manning et al., 2012), and type 2 diabetes mellitus (T2DM; Bonàs-Guarch et al., 2018), respectively. Then we excluded the potential pleiotropic SNPs we found in the last step in each analysis, and the respective SNPs with no significant differences were included in the further additional MR analysis between plasma Hcy level and SAS. The methods of all additional MR analysis were random-effects IVW method.

## Results

### Plasma Hcy Level Was Associated With Ischemic Stroke Caused by Small Artery Occlusion

**Figure 2:**
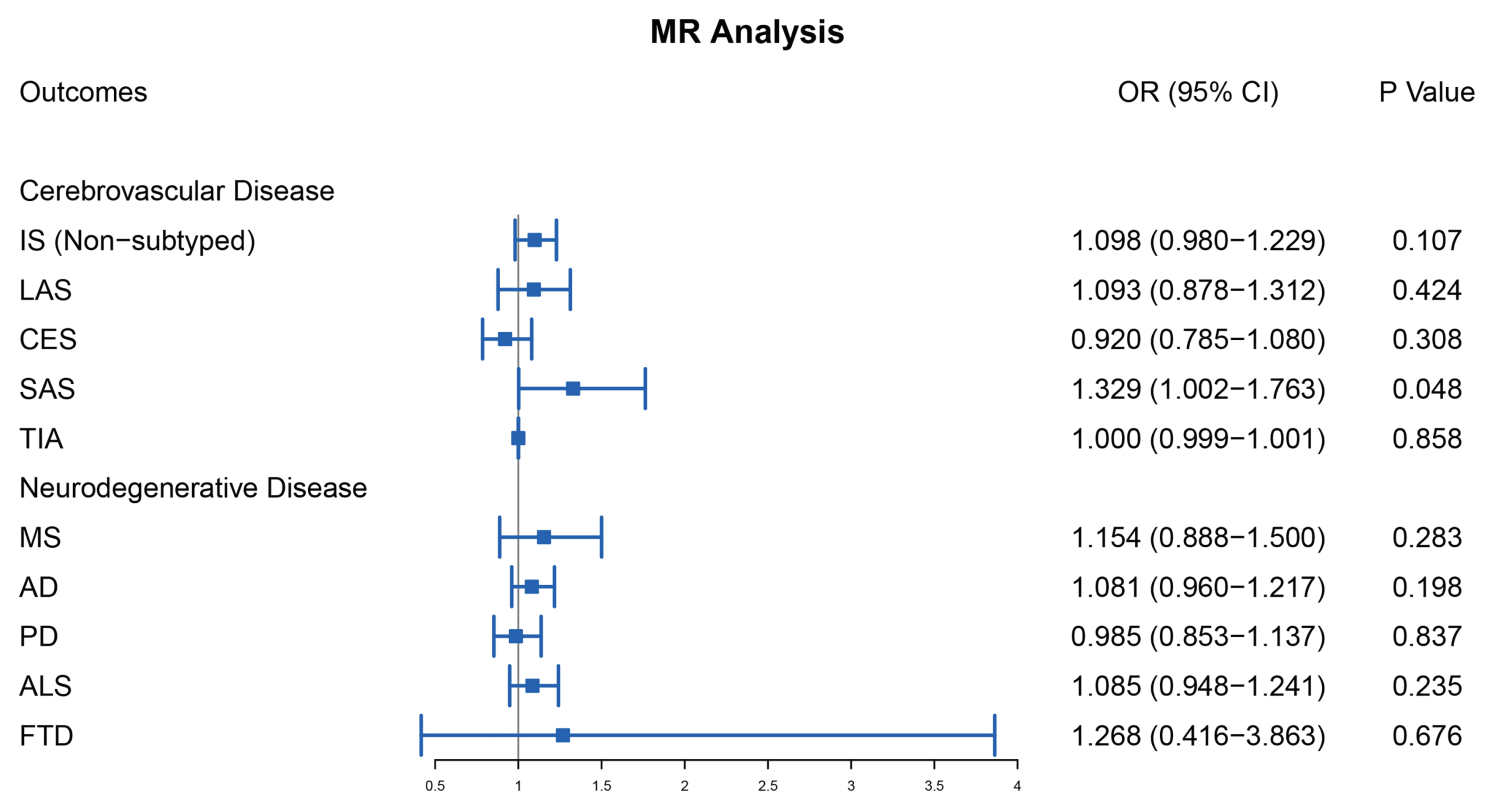
Mendelian randomization analysis of the association between plasma Hcy level and cerebrovascular and neurodegenerative diseases. OR, odds ratio; CI, confidential interval; IS, ischemic stroke; LAS, large artery atherosclerosis stroke; CES, cardioembolism stroke; SAS, small artery occlusion stroke; TIA, transient ischemic attack; MS, multiple sclerosis; AD, Alzheimer’s disease; PD, Parkinson’s disease; ALS, amyotrophic lateral sclerosis; FTD, frontotemporal dementia.

For cerebrovascular disease, we have included 13 genetic variants in most studies in addition to TIA (10 SNPs in addition to rs7422339, rs7130284, and rs548987). The result of the main MR analysis is shown in Table 1 and Figure 2. We found a causal link between plasma Hcy level and SAS (OR = 1.329, 95% CI 1.002–1.763, *p* = 0.048) but no causal link on TIA (OR = 1.000, 95% CI 0.999–1.001, *p* = 0.858) or LAS (OR = 1.093, 95% CI 0.878–1.312, *p* = 0.424), CES (OR = 0.920, 95% CI 0.785–1.080, *p* = 0.308), and nonsubtyped (OR = 1.098, 95% CI 0.980–1.229, *p* = 0.107). However, the causal link was not strong after multiple comparisons (corrected *p* = 0.005).

**Table 1:** Main MR results of the effect of Hcy on cerebrovascular disease.

SNP	Nearby gene	Nonsubtyped IS		LAA		SAO		CE		TIA	
		OR (95% CI)	*p*	OR (95% CI)	*p*	OR (95% CI)	*p*	OR (95% CI)	*p*	OR (95% CI)	*p*
rs1801133	MTHFR	1.02 (1.00–1.04)	0.352	1.00 (0.95–1.06)	0.945	1.09 (1.05–1.13)	8.37 × 10^−6^	1.00 (0.96–1.04)	0.928	1.00 (1.00–1.00)	0.930
rs2275565	MTR	1.01 (0.99–1.04)	0.211	1.02 (0.96–1.08)	0.508	1.00 (0.96–1.04)	0.990	1.05 (1.01–1.09)	0.018	NA	NA
rs9369898	MUT	1.03 (1.01–1.05)	0.001	1.02 (0.97–1.07)	0.400	1.06 (1.02–1.09)	8.32 × 10^−4^	1.01 (0.98–1.05)	0.419	1.00 (1.00–1.00)	0.056
rs7130284	NOX4	1.02 (0.99–1.06)	0.237	1.00 (0.91–1.10)	0.941	1.02 (0.97–1.07)	0.483	0.99 (0.94–1.05)	0.839	NA	NA
rs154657	DPEP1	1.01 (0.99–1.02)	0.604	1.06 (1.00–1.12)	0.043	0.98 (0.94–1.03)	0.381	1.00 (0.96–1.04)	0.894	1.00 (1.00–1.00)	0.230
rs234709	CBS	1.00 (0.98–1.02)	0.782	1.02 (0.96–1.07)	0.582	1.00 (0.96–1.04)	0.939	0.98 (0.94–1.02)	0.305	1.00 (1.00–1.00)	1.000
rs4660306	MMACHC	0.99 (0.97–1.01)	0.315	1.00 (0.95–1.05)	0.889	0.99 (0.96–1.04)	0.790	1.02 (0.98–1.05)	0.409	1.00 (1.00–1.00)	0.140
rs548987	SLC17A3	1.01 (0.98–1.03)	0.670	1.08 (1.01–1.17)	0.034	1.06 (1.01–1.11)	0.020	0.96 (0.91–1.01)	0.134	NA	NA
rs42648	GTPB10	0.98 (0.97–1.00)	0.076	1.00 (0.95–1.05)	0.972	0.99 (0.95–1.02)	0.443	1.00 (0.97–1.04)	0.984	1.00 (1.00–1.00)	0.900
rs1801222	CUBN	1.02 (1.00–1.04)	0.051	1.01 (0.96–1.07)	0.628	1.04 (1.00–1.08)	0.031	1.03 (0.99–1.07)	0.667	1.00 (1.00–1.00)	0.430
rs2251468	HNF1A	1.01 (0.99–1.03)	0.423	1.01 (0.96–1.06)	0.728	0.97 (0.94–1.01)	0.120	1.01 (0.97–1.04)	0.682	1.00 (1.00–1.00)	0.840
rs838133	FUT2	0.99 (0.97–1.01)	0.398	0.96 (0.91–1.02)	0.180	0.95 (0.91–0.99)	0.022	1.03 (0.99–1.07)	0.147	1.00 (1.00–1.00)	0.260
rs12780845	CUBN	0.99 (0.97–1.01)	0.460	1.03 (0.98–1.09)	0.219	0.99 (0.96–1.03)	0.675	1.01 (0.97–1.05)	0.560	1.00 (1.00–1.00)	1.000

SNP, single nucleotide polymorphism; OR, odds ratio; 95% CI, 95% confidential interval; IS, ischemic stroke; LAA, large artery atherosclerosis; SAO, small artery occlusion; CE, cardioembolism; TIA, transient ischemic attack; NA, not applicable.

In the sensitivity analysis (Supplementary Table S2), our result indicated strong heterogeneities of the genetic variants for SAS (*p* = 0.011) and nonsubtyped IS (*p* = 0.042). We have not found any heterogeneities of plasma Hcy level and LAS (*p* = 0.291), CES (*p* = 0.301), and TIA (*p* = 0.416). By leave-one-out analysis (Supplementary Figure S1), we found that six SNPs (rs154657, rs7130284, rs234709, rs42648, rs1801222, and rs2275565) may affect the link.

Through the MR-Egger analysis (Supplementary Table S2), there was no evidence of pleiotropy SAS (MR-Egger intercept = 0.007, 95% CI 0.038–0.053, *p* = 0.758). Also, there was no evidence of pleiotropy for LAS (MR-Egger intercept = 0.001, 95% CI 0.034–0.037, *p* = 0.953), CES (MR-Egger intercept = −0.020, 95% CI 0.043–0.003, *p* = 0.110), and nonsubtyped IS (MR-Egger intercept = −0.004, 95% CI −0.022 to 0.022, *p* = 0.680) and TIA (MR-Egger intercept = 0.0001, 95% CI −3.91 × 10^−5^ to 2.65 × 10^−4^, *p* = 0.183).

**Figure 3:**
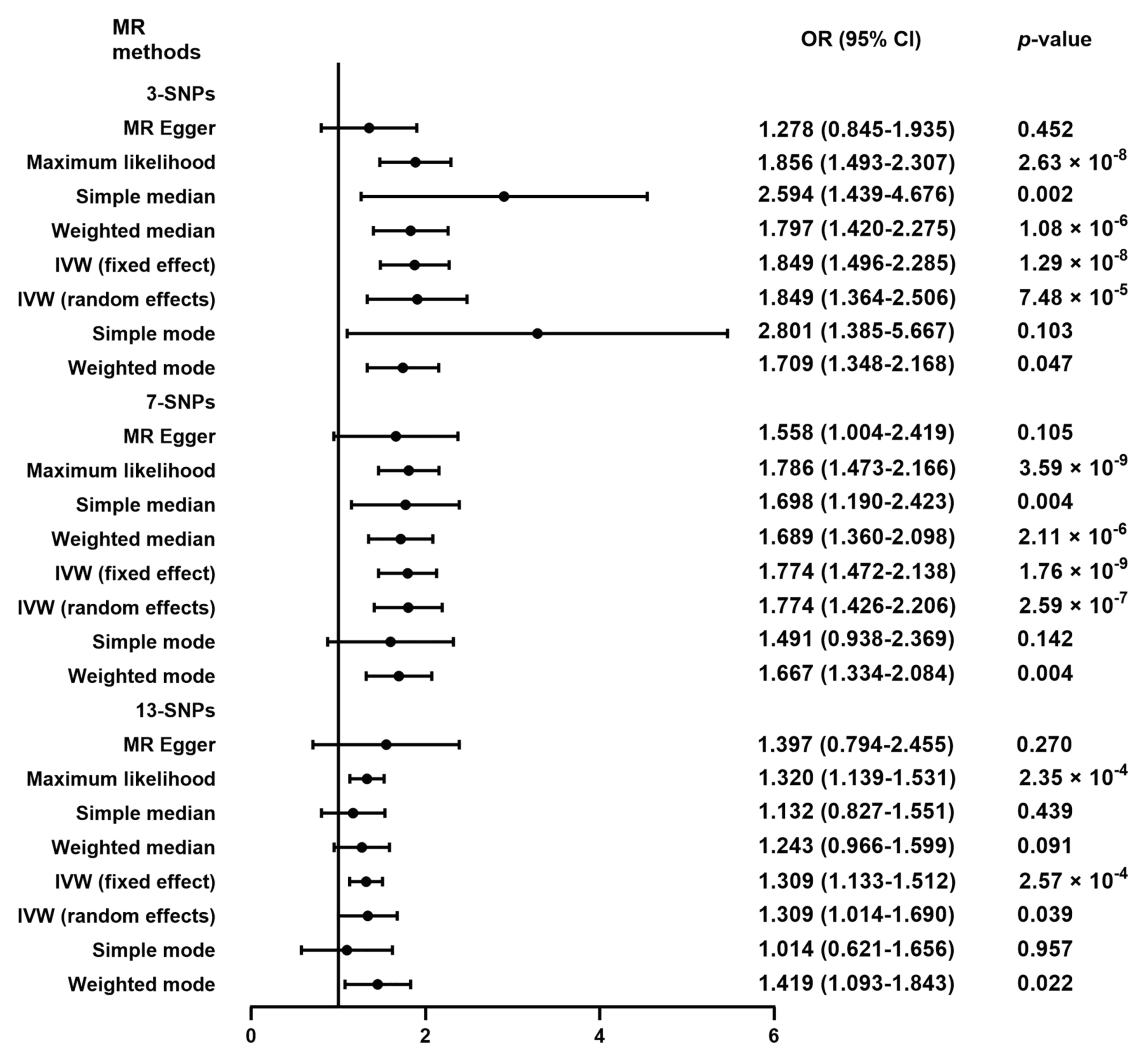
Mendelian randomization analysis of plasma Hcy level on the risk of ischemic stroke caused by small artery occlusion. OR, odds ratio; CI, confidential interval; MR, Mendelian randomization; SNPs, single nucleotide polymorphisms; IVW, inverse variance-weighted. 3-SNPs: rs1801133, rs9369898, and rs548987. 7-SNPs: rs1801133, rs9369898, rs548987, rs4660306, rs2251468, rs838133, and rs12780845. 13-SNPs: rs1801133, rs9369898, rs548987, rs4660306, rs2251468, rs838133, rs12780845, rs2275565, rs7130284, rs154657, rs234709, rs42648, and rs1801222.

As shown in Figure 3, the additional analysis indicated that a causal link between plasma Hcy level and SAS was observed using 3-SNPs (OR = 1.849, 95% CI 1.364–2.506, *p* = 7.48 × 10^−5^) and 7-SNPs (OR = 1.774, 95% CI 1.426–2.206, *p* = 2.59 × 10^−7^). Moreover, the causal link was still strong after multiple comparisons by Bonferroni correction (corrected value of *p* for 3-SNPs: 0.017 and corrected value of *p* for 7-SNPs: 0.007). In the sensitivity analyses (Supplementary Table S2), there was no evidence of significant associations observed for both 3-SNPs (*p* = 0.127) and 7-SNPs (*p* = 0.224), and there was no evidence of pleiotropy for IS caused by SAS (3-SNPs: MR-Egger intercept = 0.044, 95% CI 0.002–0.086, *p* = 0.291; 7-SNPs: MR-Egger intercept = 0.012, 95% CI −0.022 to 0.045, *p* = 0.529). Leave-one-out analysis showed that the causal link between plasma Hcy level and SAS was not substantially driven by any individual SNP (Supplementary Figure S3).

**Figure 4:**
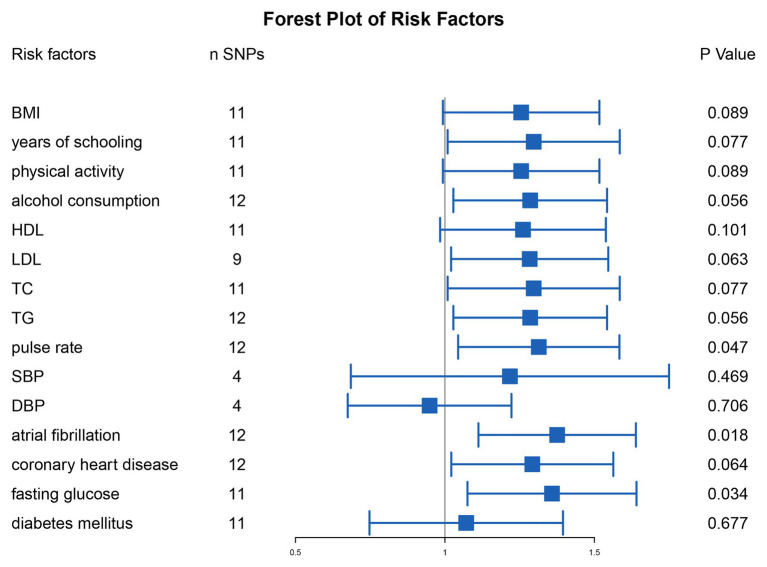
Mendelian randomization analysis of plasma Hcy level on the risk of ischemic stroke caused by small artery occlusion after exclusion for the SNPs with significant differences of the known risk factors of ischemic stroke. SNPs, single nucleotide polymorphisms; BMI, body mass index; HDL, high-density lipoprotein cholesterol; LDL, low-density lipoprotein cholesterol; TC, total cholesterol; TG, triglycerides; SBP, systolic blood pressure; DBP, diastolic blood pressure.

To analyze the causal link between Hcy and SAS deeply, first, we performed two-sample MR analysis of plasma Hcy level and the known risk factors for IS (including BMI, years of schooling, etc.), respectively, to find out the SNP with significant differences, which were considered as potential pleiotropic SNP (Supplementary Table S3). We then excluded potential pleiotropic SNPs and included SNPs with no significant differences. The results indicated that after the multipotent SNPs were excluded, SBP (OR = 1.218, 95% CI 0.685–1.750, *p* = 0.469), DBP (OR = 0.949, 95% CI 0.675–1.223, *p* = 0.706), and T2DM (OR = 1.071, 95% CI 0.748–1.395, *p* = 0.677) were potential influencers of the link between Hcy and SAS (Figure 4).

### Plasma Hcy Level Was Not Associated With Neurodegenerative Disease

For neurodegenerative disease, 13 genetic variants were included in most studies in addition to MS (6 SNPs, namely rs2275565, rs548987, rs7130284, rs154657, rs22251468, and rs12780845) and FTD (6 SNPs, namely rs9369898, rs154657, rs42648, rs12780845, rs4660306, and rs7130284). The outcomes of the main MR analysis are shown in Table 2 and Figure 2. We have not identified a significant causal link between plasma Hcy level and MS (OR = 1.154, 95% CI 0.888–1.500, *p* = 0.283), AD (OR = 1.081, 95% CI 0.960–1.217, *p* = 0.198), PD (OR = 0.985, 95% CI 0.853–1.137, *p* = 0.837), ALS (OR = 1.085, 95% CI 0.948–1.241, *p* = 0.235), and FTD (OR = 1.268, 95% CI 0.416–3.863, *p* = 0.676). By forest plot (Supplementary Figure S2), we neither have not found any SNP as a risk factor for neurodegenerative disease.

**Table 2:** Main MR results of the effect of Hcy on neurodegenerative disease.

SNP	Nearby gene	MS^†^		AD		PD		ALS		FTD^‡^	
		OR (95% CI)	*p*	OR (95% CI)	*p*	OR (95% CI)	*p*	OR (95% CI)	*p*	OR (95% CI)	*p*
rs1801133	MTHFR	NA	NA	1.01 (0.98–1.04)	0.352	1.01 (0.97–1.04)	0.698	1.02 (0.99–1.05)	0.123	NA	NA
rs2275565	MTR	0.96 (0.92–1.00)	0.059	1.00 (0.97–1.04)	0.970	1.01 (0.97–1.05)	0.591	0.98 (0.95–1.01)	0.165	NA	NA
rs9369898	MUT	NA	NA	1.02 (0.99–1.05)	0.260	1.00 (0.96–1.03)	0.882	1.00 (0.98–1.03)	0.767	0.94 (0.82–1.08)	0.356
rs7130284	NOX4	0.98 (0.92–1.04)	0.528	0.97 (0.92–1.02)	0.269	0.97 (0.89–1.06)	0.511	0.96 (0.92–1.02)	0.176	1.25 (0.96–1.64)	0.099
rs154657	DPEP1	1.01 (0.98–1.04)	0.616	1.01 (0.98–1.04)	0.550	1.00 (0.97–1.04)	0.987	1.02 (0.99–1.04)	0.263	1.13 (0.99–1.29)	0.080
rs234709	CBS	NA	NA	1.00 (0.97–1.03)	0.944	1.01 (0.97–1.05)	0.730	1.00 (0.98–1.03)	0.837	NA	NA
rs4660306	MMACHC	NA	NA	1.01 (0.98–1.04)	0.394	1.02 (0.98–1.06)	0.253	1.02 (1.00–1.05)	0.103	1.04 (0.90–1.20)	0.588
rs548987	SLC17A3	1.04 (0.99–1.09)	0.088	0.98 (0.94–1.02)	0.325	1.01 (0.95–1.06)	0.846	1.03 (0.99–1.07)	0.212	NA	NA
rs42648	GTPB10	NA	NA	0.99 (0.96–1.02)	0.390	0.98 (0.95–1.02)	0.343	0.98 (0.95–1.00)	0.087	1.03 (0.90–1.18)	0.710
rs1801222	CUBN	NA	NA	1.00 (0.97–1.03)	0.895	1.01 (0.97–1.05)	0.587	1.02 (0.99–1.05)	0.183	NA	NA
rs2251468	HNF1A	1.00 (0.96–1.03)	0.870	1.01 (0.98–1.04)	0.606	0.99 (0.96–1.02)	0.526	0.98 (0.95–1.01)	0.189	NA	NA
rs838133	FUT2	NA	NA	0.98 (0.95–1.01)	0.196	1.02 (0.98–1.06)	0.301	1.02 (0.99–1.05)	0.266	NA	NA
rs12780845	CUBN	1.02 (0.98–1.05)	0.303	0.99 (0.96–1.02)	0.377	0.99 (0.96–1.03)	0.765	1.01 (0.98–1.04)	0.616	1.04 (0.90–1.20)	0.595

SNP, single nucleotide polymorphism; OR, odds ratio; 95% CI, 95% confidential interval; MS, multiple sclerosis; AD, Alzheimer’s disease; PD, Parkinson’s disease; ALS, amyotrophic lateral sclerosis; FTD, frontotemporal dementia; NA, not applicable.

† Included SNPs: rs2275565 (proxy: rs10158822), rs7130284 (proxy: rs11018628), rs154657 (proxy: rs460879), rs548987 (proxy: rs501220), rs2251468 (proxy: rs2244608), and rs12780845 (proxy: rs10490958).

‡ Included SNPs: rs9369898 (proxy: rs2501968), rs7130284 (proxy: rs10501705), rs154657 (proxy: rs460879), rs4660306 (proxy: rs2991966), rs42648 (proxy: rs42659), and rs12780845 (proxy: rs7095324).

As shown in Supplementary Table S2, the sensitivity analysis indicated no heterogeneities among individual SNPs (MS: *p* = 0.236; AD: *p* = 0.857; PD: *p* = 0.856; ALS: *p* = 0.129; FTD: *p* = 0.213), and there was no evidence of pleiotropy for the outcomes of neurodegenerative disease (MS: MR-Egger intercept = −0.0002, 95% CI −0.065 to 0.065, *p* = 0.996; AD: MR-Egger intercept = −0.005, 95% CI −0.024 to 0.013, *p* = 0.593; PD: MR-Egger intercept = −0.016, 95% CI −0.038 to 0.007, *p* = 0.206; ALS: MR-Egger intercept = −0.021, 95% CI −0.040 to −0.002, *p* = 0.053; FTD: MR-Egger intercept = 0.014, 95% CI −0.188 to 0.217, *p* = 0.897). The leave-one-out sensitivity analysis suggested that all MR analysis results for neurodegenerative disease were not driven dramatically by any single SNP (Supplementary Figure S1).

## Discussion

In our two-sample MR study, there was little evidence of the causal link between high plasma Hcy and high risk of SAS. However, after multiple comparisons, no statistically significant impact was found. Additional analysis showed strong evidence by both 3-SNP and 7-SNP methods. We also found that SBP, DBP, and T2DM were potential influencers in the link between Hcy and SAS. For other types of IS, genetically higher plasma Hcy level was not associated with higher risk of LAS, CES, and nonsubtyped. In addition, our results showed no causal links between plasma Hcy level and neurodegenerative disease.

Previous studies have reported the link between HHcy and atherosclerotic vascular disease and neurodegenerative disease (Hankey and Eikelboom, 2001; Morris, 2003; Zhang et al., 2018). Some studies demonstrated that Hcy contributed to endothelial dysfunction by oxidative stress, DNA damage, and apoptosis (Currò et al., 2014). Hcy could induce an inflammatory environment by upregulating inflammatory factors, including C-reactive protein and intracellular adhesion molecule-1 (Durga et al., 2005). Because of the high susceptibility to disease of cerebral small vessels, the harmful effect of Hcy can be more obvious (Wardlaw et al., 2019).

Epidemiologic observational studies have demonstrated Hcy as a risk factor for cardiovascular disease. A meta-analysis including 10 studies detected a significant dose–response association of Hcy with the risk of IS (Wu et al., 2020). Previous studies also reported that HHcy was associated with microbleeds and leukoaraiosis (Feng et al., 2013; Yoo et al., 2020). By profiling the genetic variant MTHFR C677T among IS with different origins, Rutten-Jacobs et al. (2016) reported that MTHFR C677T was associated with lacune and higher white hyperintensity, but not LAS or CES, which partly explained the uncertainty about the efficacy of lowering Hcy treatment for stroke patients. A meta-analysis including 13 case–control studies of Chinese patients reported that all subtypes of IS had higher plasma Hcy than healthy controls (Zhang et al., 2020). Previous MR studies have reported the association between Hcy and cardiovascular disease. Casas et al. found a causal link between Hcy and IS by profiling MTHFR C677T polymorphism (Casas et al., 2005). MTHFR gene mutations were associated with lacunes and cerebral atrophy, too (Cao et al., 2020). An MR study investigating the level of Hcy, folate, and B vitamins and IS or CHD reported a similar result (Larsson et al., 2019). Our main analysis had a consistent result. A possible mechanism of the link between Hcy and SAS was the small vessels’ higher sensitivity to stimulus like high pressure and oxidative stress (Wardlaw et al., 2019). In addition, HHcy was associated with endothelial dysfunction, thus leading to blood–brain barrier (BBB) dysfunction and even disruption (Nam et al., 2019). The disruption of the BBB and chronic hypoperfusion finally cause the development of white matter hyperintensity, cerebral microbleeds, and enlarged perivascular space (Nam et al., 2019). We found that, after multiple comparisons, the effect was not strong. But through an additional analysis using 3-SNP and 7-SNP methods, even after the correction, the effect was still strong. Thus, some candidate risk genes, like MTHFR, which provided a novel monogenic cause of SAS, still require further investigation, and the mechanism needs to be further clarified.

Another main concern is the use of lowering Hcy medication. HHcy was relatively common in atherosclerotic cardiovascular disease patients. A meta-analysis demonstrated a linear inverse link between dietary intake of Hcy metabolism-related B vitamins (Chen et al., 2020). However, the rate of use of folate or B vitamin supplementation for lowering Hcy was not as high as expected partly due to the controversial results of previous clinical trials. The VITAmins TO Prevent Stroke (VITATOPS) trial indicated that B vitamin supplementation was a protective factor for SAS but not for other subtypes (VITATOPS Trial Study Group, 2010), and a substudy of VITATOPS showed that a 2-year B vitamin medication could significantly reduce the volume of white matter hyperintensities (Cavalieri et al., 2012). Besides, the China Stroke Primary Prevention Trial concluded that folic acid supplementation could significantly reduce the risk of first stroke among subjects with hypertension (Huo et al., 2015). Therefore, lowering Hcy treatment may benefit individuals with specific origins of cardiovascular disease. For neurodegeneration, most clinical trials obtained negative results of lowering Hcy treatment (Chen et al., 2016; Kwok et al., 2020). Here, we performed an additional analysis by excluding the potential pleiotropic SNPs, and our results indicated that the link between genetically high Hcy and SAS could be influenced by the multipotent capacity of the Hcy-related genetic variants, particularly by the effect on blood pressure and T2DM for the first time by the MR method. The effect may originate from both horizontal and vertical pleiotropy of the genetic variants. Perhaps the genetic variants associated with Hcy influence blood pressure and T2DM or Hcy affects the risk of SAS through blood pressure and T2DM. This effect can affect the occurrence of SAS and the therapeutic efficacy of lowering Hcy. SAS, T2DM, and hypertension all show small vessel lesions and the long-term metabolic disturbance is associated with dysfunction of the endothelium and activation and irritation of the inflammatory environment, which were potential mechanisms of SAS. Therefore, lowering Hcy treatments could be considered for subjects with HHcy and metabolic syndrome including hypertension and T2DM. Moreover, clinical trials will focus on the diversity and accuracy of lowering Hcy treatment and find out the population who benefit from it.

In addition to the adverse effect on vasculature, previous studies have shown that HHcy was associated with neurodegeneration, so we tried to find the link between Hcy and neurodegeneration. The toxicity of Hcy on neurons may contribute to the accumulation of β-amyloid, calcium influx, and apoptosis of neurons, making subjects more prone to develop dementia (Obeid and Herrmann, 2006). Despite several studies reporting the links between Hcy and neurodegenerative disease (Ostrakhovitch and Tabibzadeh, 2019), we have not found any evidence of the link between Hcy and AD from our results, which was consistent with a previous MR study (Larsson et al., 2017). We also found no causal link between Hcy and other neurodegenerative disease, including PD, ALS, MS, and FTD. Therefore, the common pathogenesis of SAS and dementia remains to be validated and investigated.

Our MR study still has some limitations. First of all, one major limitation of MR is the bias because of pleiotropy, indicating one genetic variant influences various phenotypes. Moreover, we can hardly exclude that all the SNPs in our study probably had an impact on the risk of cerebrovascular or neurodegenerative disease through other mechanisms except for influencing plasma Hcy level. Despite that we have not found any evidence of pleiotropy in the MR-Egger intercept analysis, this result may be hindered by a relatively low number of SNPs, and a low number of SNPs may overestimate the effect of exposure on the outcome. Therefore, more SNPs associated with plasma Hcy level and exposures including IS need to be identified in studies with a larger sample size with higher resolution. Besides, some risk factors for SAS, including hypertension, dyslipidemia, diabetes mellitus, smoking, and obesity, are risk factors for other subtypes of stroke as well. Thus, such risk factors may partially influence plasma Hcy level or contribute to the effect of Hcy on SAS *via* other mechanisms. Despite having excluded the SNPs with such properties in an additional analysis, fewer SNPs were included and a multivariable MR analysis may present a more meaningful suggestion. Finally, our MR study was originated from subjects with European ancestry, so population stratification inevitably existed. As a result, the conclusion is not supposed to be generalized to other ethnicities around the globe.

## Conclusion

Through a two-sample MR study, we found that there was a causal link between plasma Hcy level and SAS. Another additional analysis indicated that SBP, DBP, and T2DM serve as influencers in this association. However, no causal links were identified between Hcy and other subtypes of IS, TIA, or neurodegenerative disease. For the prevention of IS, patients with T2DM or hypertension may benefit more from lowering Hcy treatment. Revealing the underlying common pathways of HHcy, hypertension, T2DM, and SAS would be of importance.

## Data Availability Statement

The original contributions presented in the study are included in the article/Supplementary Material, further inquiries can be directed to the corresponding authors.

## Ethics Statement

Written informed consent was obtained from the individual(s) for the publication of any potentially identifiable images or data included in this article.

## Author Contributions

ZX and YX conceived and designed the study. SL, CL, and YT collected the data. WL and LZ analyzed the data. BS, ZX, and YX were involved in the supervision of the study. HF, RZ, and BS were involved in the interpretation of the data. WL drafted the manuscript. All authors contributed to the article and approved the submitted version.

### Conflict of Interest

The authors declare that the research was conducted in the absence of any commercial or financial relationships that could be construed as a potential conflict of interest.The reviewer YP declared a past co-authorship with the authors HF, BS and YX to the handling editor.

## Supplementary Material

Supplementary Figure S1 Leave-one-out analysis of the effect of plasma Hcy level on cerebrovascular and neurodegenerative disease. IS, ischemic stroke; LAS, large artery atherosclerosis stroke; CES, cardio-embolism stroke; SAS, small artery occlusion stroke; TIA, transient ischemic attack; MS, multiple sclerosis; AD, Alzheimer’s Disease; PD, Parkinson’s disease; ALS, amyotrophic lateral sclerosis; FTD, frontotemporal dementia. The dot and the bar indicated the estimates and 95% CI when the specific SNP is removed.

Supplementary Figure S2 Forest plot of the effect of plasma Hcy level on cerebrovascular and neurodegenerative disease. IS, ischemic stroke; LAS, large artery atherosclerosis stroke; CES, cardio-embolism stroke; SAS, small artery occlusion stroke; TIA, transient ischemic attack; MS, multiple sclerosis; AD, Alzheimer’s Disease; PD, Parkinson’s disease; ALS, amyotrophic lateral sclerosis; FTD, frontotemporal dementia. The dot and the bar indicated the causal estimate of plasma Hcy level on risk of cerebrovascular and neurodegenerative disease.

Supplementary Figure S3 Scatter plot and leave-one-out analysis of the effect of plasma Hcy level on ischemic stroke caused by small artery occlusion. MR, Mendelian randomization; SNPs, single nucleotide polymorphisms; Hcy, homocysteine; SAS, small artery occlusion stroke. **(A)** Scatter plot: each black dot indicated a SNP, plotted by the estimate of SNP on plasma Hcy level and the estimate of SNP on the risk of ischemic stroke caused by small artery occlusion with standard error bars. **(B)** Leave-one-out analysis: the dot and the bar indicated the estimates and 95% CI when the specific SNP is removed. 3-SNPs: rs1801133, rs9369898, and rs548987. 7-SNPs: rs1801133, rs9369898, rs548987, rs4660306, rs2251468, rs838133, and rs12780845. 13-SNPs: rs1801133, rs9369898, rs548987, rs4660306, rs2251468, rs838133, rs12780845, rs2275565, rs7130284, rs154657, rs234709, rs42648, and rs1801222.
