# Supplementary material for: A Mendelian Randomization Study of Plasma Homocysteine Levels and Cerebrovascular and Neurodegenerative Diseases
Source: Front Genet. 2021 Apr 1;12:653032. doi: 10.3389/fgene.2021.653032 (PMC8047106; doi:10.3389/fgene.2021.653032)
Supplement: Supplementary file 1 [file Table_1.DOCX]

Supplementary Table 1. SNPs included associated with plasma Hcy level.

| SNP | Nearby  Gene | EA | OA | EAF | β | SE | *p*-value |
| --- | --- | --- | --- | --- | --- | --- | --- |
| rs1801133 | MTHFR | A | G | 0.34 | 0.1583 | 0.007 | 4.34 × 10^-104^ |
| rs2275565 | MTR | T | G | 0.21 | -0.0542 | 0.009 | 1.96 × 10^-10^ |
| rs9369898 | MUT | A | G | 0.62 | 0.0449 | 0.007 | 2.17 × 10^-10^ |
| rs7130284 | NOX4 | C | T | 0.07 | 0.1242 | 0.013 | 1.88 × 10^-20^ |
| rs154657 | DPEP1 | A | G | 0.47 | 0.0963 | 0.007 | 1.74 ×10^-43^ |
| rs234709 | CBS | T | C | 0.45 | -0.0718 | 0.007 | 3.90 × 10^-24^ |
| rs4660306 | MMACHC | C | T | 0.33 | -0.0435 | 0.007 | 2.33 × 10^-09^ |
| rs548987 | SLC17A3 | C | G | 0.13 | 0.0597 | 0.010 | 1.12 × 10^-08^ |
| rs42648 | GTPB10 | G | A | 0.40 | 0.0395 | 0.007 | 1.97 × 10^-08^ |
| rs1801222 | CUBN | G | A | 0.34 | -0.0453 | 0.007 | 8.43 × 10^-10^ |
| rs2251468 | HNF1A | A | C | 0.65 | -0.0512 | 0.007 | 1.28 × 10^-12^ |
| rs838133 | FUT2 | G | A | 0.45 | -0.0422 | 0.007 | 7.48 × 10^-09^ |
| rs12780845 | CUBN | G | A | 0.65 | -0.0529 | 0.009 | 7.80 × 10^-10^ |

SNP: single nucleotide polymorphism, EA: effect allele, OA: other allele, EAF: effect allele frequency, SE: standardized error.
