# Supplementary material for: A Mendelian Randomization Study of Plasma Homocysteine Levels and Cerebrovascular and Neurodegenerative Diseases
Source: Front Genet. 2021 Apr 1;12:653032. doi: 10.3389/fgene.2021.653032 (PMC8047106; doi:10.3389/fgene.2021.653032)
Supplement: Supplementary file 2 [file Table_2.DOCX]

Supplementary Table 2. Sensitivity tests of plasma Hcy level and cerebrovascular and neurodegenerative disease.

|  | Pleiotropy | |  | Heterogeneity | |
| --- | --- | --- | --- | --- | --- |
|  | Intercept | *p*-value |  | Q | *p*-value |
| Outcomes |  |  |  |  |  |
| IS | -0.0050 | 0.680 |  | 21.612 | 0.042 |
| LAA | 0.0011 | 0.953 |  | 13.042 | 0.366 |
| CE | -0.0204 | 0.110 |  | 14.001 | 0.301 |
| SAO | 0.0074 | 0.758 |  | 25.861 | 0.011 |
| TIA | 0.0001 | 0.183 |  | 9.232 | 0.416 |
| MS | -0.0002 | 0.996 |  | 6.805 | 0.236 |
| AD | -0.0053 | 0.593 |  | 6.239 | 0.857 |
| PD | -0.0155 | 0.206 |  | 5.073 | 0.956 |
| ALS | -0.0207 | 0.054 |  | 17.577 | 0.129 |
| FTD | 0.0143 | 0.897 |  | 7.109 | 0.213 |
|  |  |  |  |  |  |
| Addition |  |  |  |  |  |
| 3-SNPs | 0.0439 | 0.291 |  | 4.125 | 0.127 |
| 7-SNPs | 0.0117 | 0.529 |  | 8.193 | 0.224 |

IS: ischemic stroke, LAA: large artery atherosclerosis, CE: cardio-embolism, SAO: small artery occlusion, TIA: transient ischemic attack, MS: multiple sclerosis, AD: Alzheimer’s disease, PD: Parkinson’s disease, ALS: amyotrophic lateral sclerosis, FTD: frontotemporal dementia.
