# Supplementary material for: A Mendelian Randomization Study of Plasma Homocysteine Levels and Cerebrovascular and Neurodegenerative Diseases
Source: Front Genet. 2021 Apr 1;12:653032. doi: 10.3389/fgene.2021.653032 (PMC8047106; doi:10.3389/fgene.2021.653032)
Supplement: Supplementary file 3 [file Table_3.DOCX]

Supplementary Table 3. Results of the Mendelian randomization analysis of plasma Hcy level and the known risk factors for ischemic stroke

| SNP | Risk factors | | | | | | | | | | | | | | | |
| --- | --- | --- | --- | --- | --- | --- | --- | --- | --- | --- | --- | --- | --- | --- | --- | --- |
|  | AF | ALC | BMI | CHD | DBP | DM | GLUC | HDL | LDL | PA | PR | SBP | YSCH | SMK | TC | TG |
| rs12780845 | 0.854 | 0.636 | 0.520 | 0.642 | 0.000^*^ | 0.351 | 0.626 | 0.261 | 0.236 | 0.250 | 0.850 | 0.000^*^ | 0.710 | 0.075 | 0.161 | 0.618 |
| rs154657 | 0.023^*^ | 0.807 | 0.190 | 0.134 | 0.000^*^ | 0.470 | 0.305 | 0.293 | 0.931 | 0.750 | 0.530 | 0.000^*^ | 0.875 | 0.910 | 0.720 | 0.259 |
| rs1801133 | 0.386 | 0.158 | 0.670 | 0.184 | 0.000^*^ | 0.021^*^ | 0.184 | 0.096 | 0.280 | 0.640 | 0.960 | 0.000^*^ | 0.143 | 0.270 | 0.635 | 0.474 |
| rs1801222 | 0.320 | 0.528 | 0.550 | 0.472 | 0.003^*^ | 0.917 | 0.320 | 0.707 | 0.039^*^ | 0.550 | 0.980 | 0.002^*^ | 0.200 | 0.081 | 0.337 | 0.159 |
| rs2251468 | 0.377 | 0.110 | 0.770 | 0.000 | 0.000^*^ | 0.015^*^ | 0.112 | 0.002^*^ | 0.000^*^ | 0.320 | 0.120 | 0.006^*^ | 0.002^*^ | 0.830 | 0.030^*^ | 0.611 |
| rs2275565 | 0.984 | 0.763 | 0.230 | 0.609 | 0.554 | 0.992 | 0.836 | 0.527 | 0.490 | 0.250 | 0.890 | 0.592 | 0.816 | 0.950 | 0.741 | 0.415 |
| rs234709 | 0.273 | 0.114 | 0.500 | 0.336 | 0.104 | 0.766 | 0.024^*^ | 0.254 | 0.071 | 0.830 | 0.430 | 0.021^*^ | 0.235 | 0.790 | 0.141 | 0.219 |
| rs42648 | 0.821 | 0.526 | 0.600 | 0.056 | 0.000^*^ | 0.123 | 0.000^*^ | 0.092 | 0.170 | 0.076 | 0.590 | 0.111 | 0.192 | 0.300 | 0.811 | 0.156 |
| rs4660306 | 0.500 | 0.848 | 0.085 | 0.943 | 0.196 | 0.348 | 0.660 | 0.124 | 0.997 | 0.790 | 0.019^*^ | 0.058 | 0.004^*^ | 0.720 | 0.029^*^ | 0.449 |
| rs548987 | 0.305 | 0.063 | 0.016^*^ | 0.722 | 0.000^*^ | 0.830 | 0.316 | 0.005^*^ | 0.000^*^ | 0.000^*^ | 0.600 | 0.097 | 0.254 | 0.300 | 0.216 | 0.944 |
| rs7130284 | 0.778 | 0.212 | 0.075 | 0.164 | 0.801 | 0.311 | 0.490 | 0.828 | 0.999 | 0.370 | 0.057 | 0.029^*^ | 0.857 | 0.370 | 0.467 | 0.634 |
| rs838133 | 0.235 | 0.001^*^ | 0.008^*^ | 0.988 | 0.000^*^ | 0.055 | 0.555 | 0.956 | 0.000^*^ | 0.000^*^ | 0.650 | 0.000^*^ | 0.424 | 0.920 | 0.128 | 0.002^*^ |
| rs9369898 | 0.127 | 0.912 | 0.098 | 0.481 | 0.013^*^ | 0.404 | 0.664 | 0.706 | 0.703 | 0.540 | 0.740 | 0.000^*^ | 0.437 | 0.580 | 0.226 | 0.648 |

^*^: p <0.05

SNP: single nucleotide polymorphism; AF: atrial fibrillation; ALC: alcohol consumption; BMI: body mass index; CHD: coronary heart disease; DBP: diastolic blood pressure; DM: type 2 diabetes mellitus; GLUC: fasting blood glucose; HDL: high-density lipoprotein cholesterol; LDL: low-density lipoprotein cholesterol; PA: physical activity; PR: pulse rate; SBP: systolic blood pressure; YSCH: years of schooling; SMK: current tobacco smoking; TC: total cholesterol; TG: triglycerides.
