# Supplementary figures and images for: A Mendelian Randomization Study of Plasma Homocysteine Levels and Cerebrovascular and Neurodegenerative Diseases
Source: Front Genet. 2021 Apr 1;12:653032. doi: 10.3389/fgene.2021.653032 (PMC8047106; doi:10.3389/fgene.2021.653032)

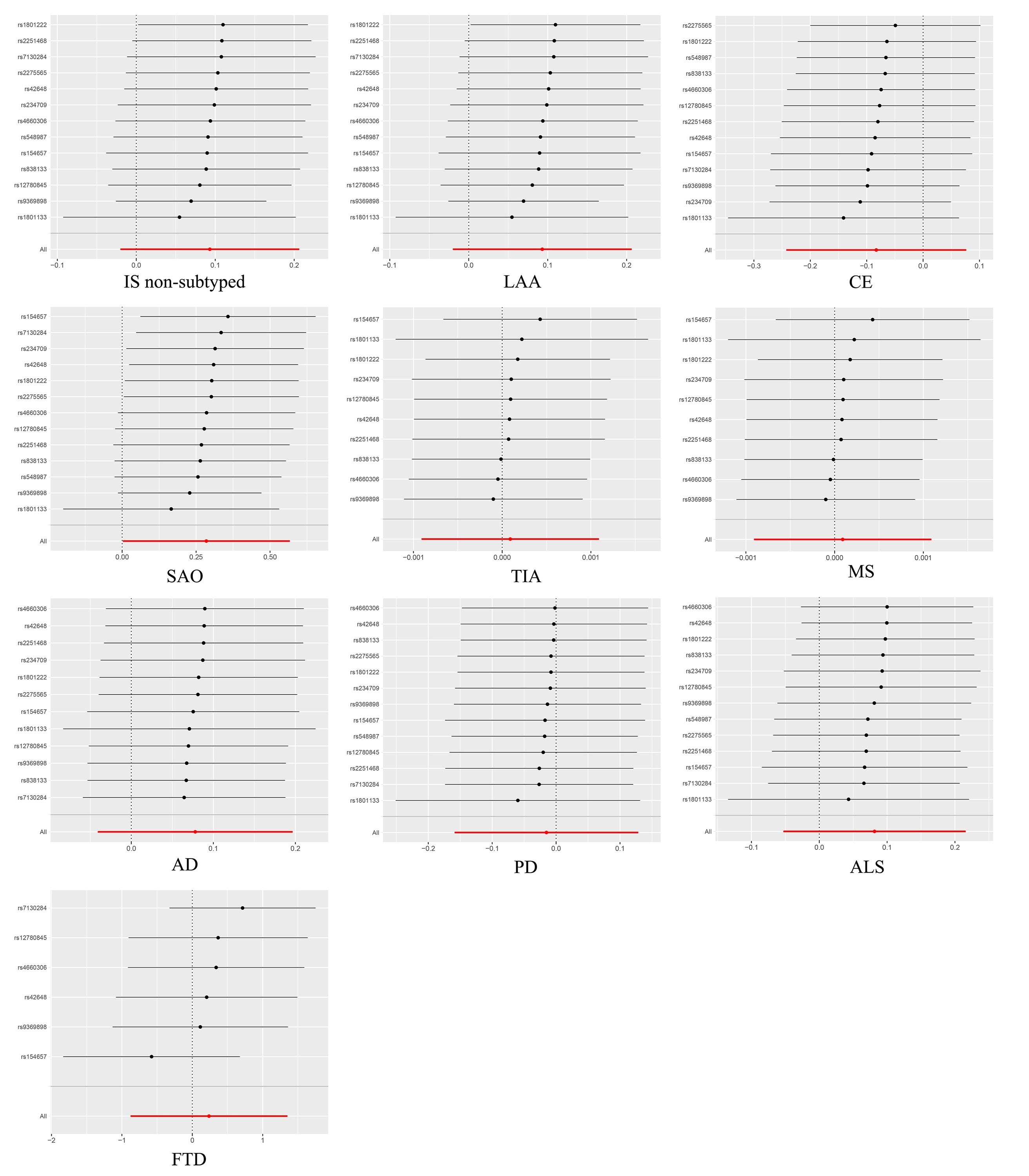

Supplement: Supplementary Figure S1 — Leave-one-out analysis of the effect of plasma Hcy level on cerebrovascular and neurodegenerative disease. IS, ischemic stroke; LAS, large artery atherosclerosis stroke; CES, cardio-embolism stroke; SAS, small artery occlusion stroke; TIA, transient ischemic attack; MS, multiple sclerosis; AD, Alzheimer’s Disease; PD, Parkinson’s disease; ALS, amyotrophic lateral sclerosis; FTD, frontotemporal dementia. The dot and the bar indicated the estimates and 95% CI when the specific SNP is removed. [file Image_1.JPEG]

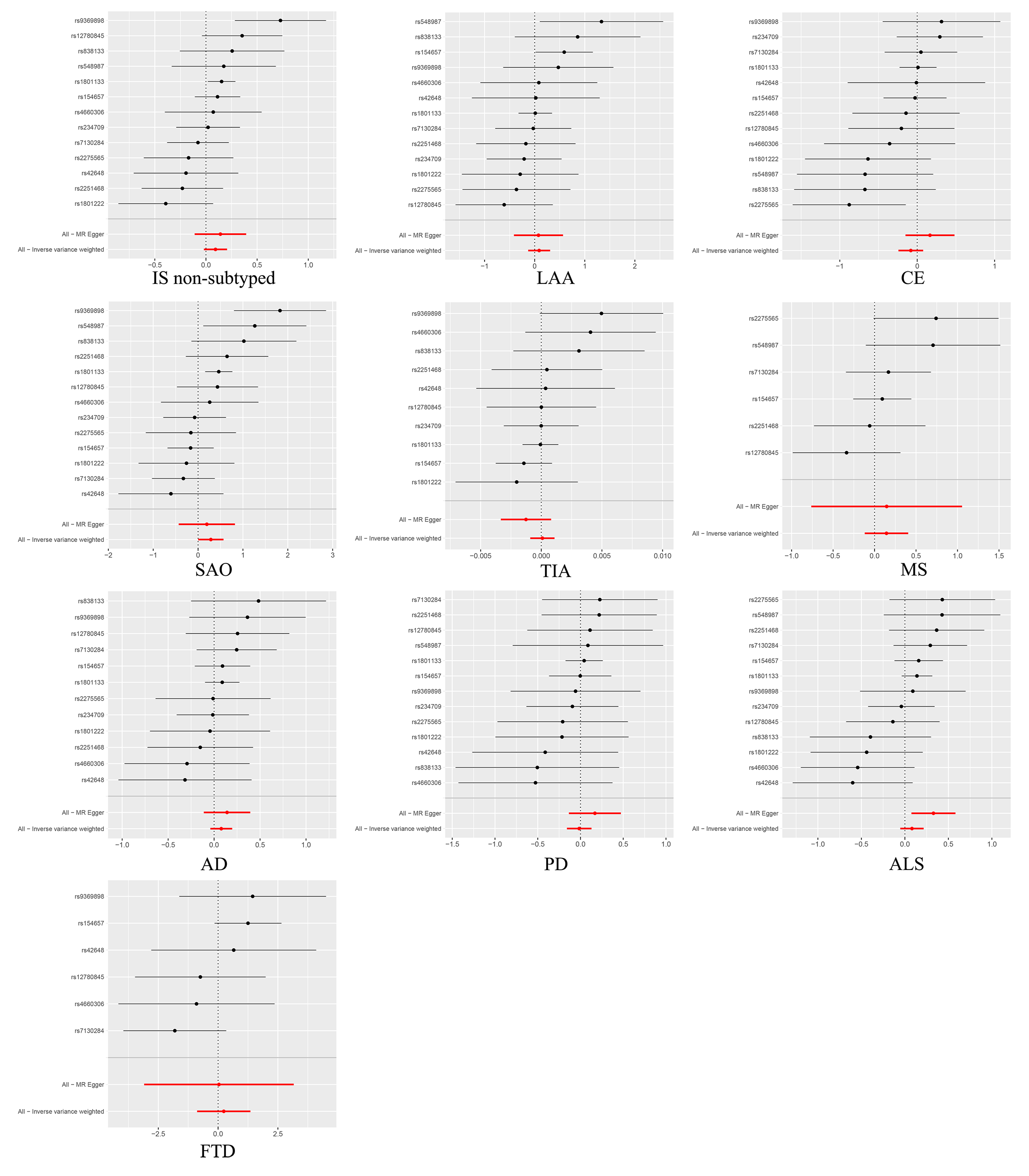

Supplement: Supplementary Figure S2 — Forest plot of the effect of plasma Hcy level on cerebrovascular and neurodegenerative disease. IS, ischemic stroke; LAS, large artery atherosclerosis stroke; CES, cardio-embolism stroke; SAS, small artery occlusion stroke; TIA, transient ischemic attack; MS, multiple sclerosis; AD, Alzheimer’s Disease; PD, Parkinson’s disease; ALS, amyotrophic lateral sclerosis; FTD, frontotemporal dementia. The dot and the bar indicated the causal estimate of plasma Hcy level on risk of cerebrovascular and neurodegenerative disease. [file Image_2.TIF]

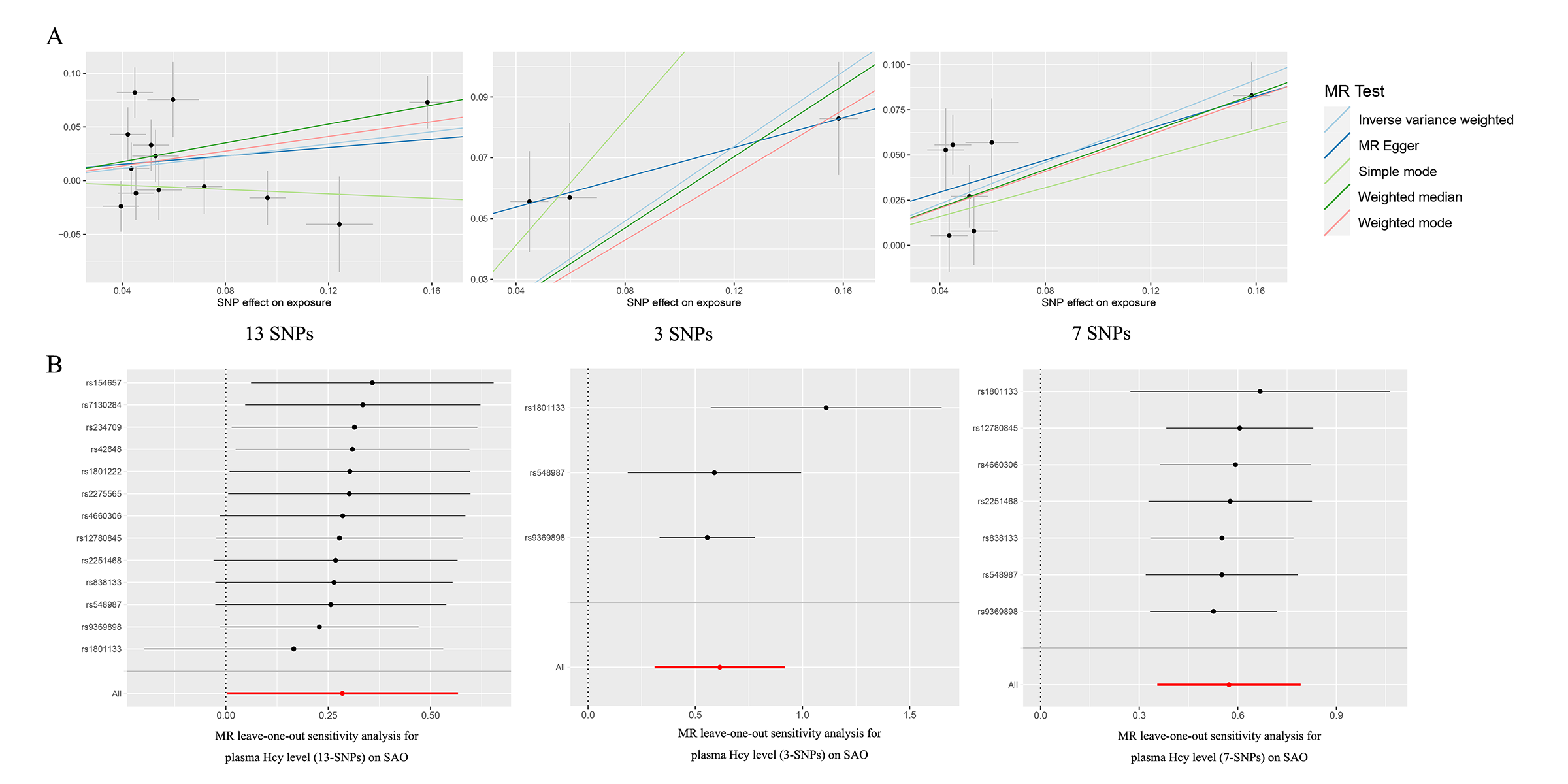

Supplement: Supplementary Figure S3 — Scatter plot and leave-one-out analysis of the effect of plasma Hcy level on ischemic stroke caused by small artery occlusion. MR, Mendelian randomization; SNPs, single nucleotide polymorphisms; Hcy, homocysteine; SAS, small artery occlusion stroke. (A) Scatter plot: each black dot indicated a SNP, plotted by the estimate of SNP on plasma Hcy level and the estimate of SNP on the risk of ischemic stroke caused by small artery occlusion with standard error bars. (B) Leave-one-out analysis: the dot and the bar indicated the estimates and 95% CI when the specific SNP is removed. 3-SNPs: rs1801133, rs9369898, and rs548987. 7-SNPs: rs1801133, rs9369898, rs548987, rs4660306, rs2251468, rs838133, and rs12780845. 13-SNPs: rs1801133, rs9369898, rs548987, rs4660306, rs2251468, rs838133, rs12780845, rs2275565, rs7130284, rs154657, rs234709, rs42648, and rs1801222. [file Image_3.TIF]
